# Changing patterns of nasopharyngeal carcinoma incidence in Hong Kong: a 30-year analysis and future projections

**DOI:** 10.1186/s12885-023-11296-1

**Published:** 2023-08-16

**Authors:** Xiaoyan Wang, Haifeng Sun, Linchang Li, Zhenhai Gan, Xiaoming Wu, Jianqiang Du

**Affiliations:** 1 The Key Laboratory of Biomedical Information Engineering of Ministry of Education, School of Life Science and Technology, Xi’an Jiaotong University, No.28, Xianning West Road, Xi’an, Shaanxi, 710049 China; 2 Third Department of Medical Oncology, Shaanxi Provincial Cancer Hospital Affiliated to Medical College of Xi’an Jiaotong University, Xi’an, Shaanxi, China; 3 Department of Clinical Medicine, Second Clinical School of Medicine, Shaanxi, University of Chinese Medicine, Xianyang, Shaanxi China

**Keywords:** Nasopharyngeal carcinoma, Incidence, Period analysis, Cohort effect, Demographic factors

## Abstract

**Background:**

This study aims to evaluate the relationship between age, period, and birth cohort with the incidence trends of Nasopharyngeal Carcinoma (NPC) in Hong Kong, make projections through 2030 and parse the drivers of the incidence.

**Methods:**

Using data from the Hong Kong Cancer Registry, we used an age-period-cohort model to uniquely estimate age, period, and cohort effects on NPC incidence trends and make projections. We further assessed the drivers of NPC incidence using a validated decomposition algorithm.

**Results:**

From 1991 to 2020, crude and age-standardized incidence rates of NPC decreased significantly. The net drifts showed significant downward trends for both sexes, and local drift declined in all age groups. Period and cohort rate ratios revealed monotonic declining patterns for both sexes. Projections suggested that NPC incidence will continue to decline. Population decomposition showed that while population growth and ageing have led to an increase in NPC cases, epidemiologic changes offset these increases, resulting in an encouraging downward trend in the incidence and new NPC cases in Hong Kong.

**Conclusions:**

The period and cohort risk of NPC in Hong Kong decreased, and epidemiologic changes offset the contribution of demographic factors, resulting in a continued decline in NPC incidence and cases.

**Supplementary Information:**

The online version contains supplementary material available at 10.1186/s12885-023-11296-1.

## Background

Nasopharyngeal carcinoma (NPC) is a rare type of cancer that originates from the lining of the nasopharyngeal mucosal [1]. According to the International Agency for Research on Cancer, there were over 130,000 new cases of NPC in 2020, representing only 0.7% of all diagnosed cancers that year [2]. However, the global geographic distribution of NPC is highly unbalanced, with a prevalence in southern China, including Hong Kong, while being uncommon in most parts of the world [3–5]. In Hong Kong, NPC is the eighth most common cancer in men and the tenth most common in women, with men aged 20–44 being the most affected group [6]. Over the past few decades, NPC incidence has significantly declined in many parts of the world, including Hong Kong [5, 7], where mean annual changes were -1.9% and -4.3% for males and females, respectively, during 2010–2019 [8].

The decline in NPC incidence in Hong Kong is likely due to reduced exposure to multiple risk factors, including Epstein-Barr virus (EBV) infection, host genetics, dietary consumption of Chinese-style salted fish, and smoking [1, 4, 5, 7, 9]. However, the prevalence of these risk factors varies over time, and the risk of developing NPC may differ between birth cohorts. Additionally, the incidence of NPC shows a single peak at around 45–59 years of age [5, 10], and population ageing and population growth may contribute to increased incidence rates. Thus, the observed trend in NPC incidence in Hong Kong over the past few decades may result from combined changes in risk factor exposure and demographic shifts.

Previous publications have documented changes in the epidemiology of NPC, but the reasons for the decline in incidence remain unclear. Therefore, in this study, we used an age-period-cohort (APC) model to investigate how patient age, calendar period of diagnosis, and birth cohort interact with NPC incidence in Hong Kong. This model allows us to separate and analyze the impact of temporal changes and birth cohort variation on NPC incidence [11–13]. We also projected the future incidence of NPC in Hong Kong through 2030 using a model that considers age, period, and cohort effects. Finally, we investigated temporal variations in new NPC cases and attributed these variations to demographic and epidemiologic shifts. Our findings are crucial for assessing the effectiveness of NPC prevention, and they can inform public policy formulation, resource allocation, and screening program design.

## Methods

### Data

We used publicly available data on NPC cases diagnosed in Hong Kong from 1991 to 2020. Data on NPC incidence among Hong Kong residents by year of diagnosis, age group, and sex were obtained from the Hong Kong Cancer Registry (HKCaR), a population-based cancer registry covering the entire population of the region [6]. NPC was coded 147 and C11 of the 9th and 10th International Classification of Diseases (ICD-9 and ICD-10), respectively. Patients under 20 were excluded due to the relatively low incidence of NPC in this age range. Population estimates from 1991 to 2030 were collected from the United Nations (UN) World Population Prospects 2019 Revision [14]. We calculated age-standardized incidence rates using the World Health Organization (WHO) standard population in 2000. According to the ethics committee policy of the School of Life Science and Technology, Xi'an Jiaotong University, this study was granted an exemption from ethical review. This exemption was based on the fact that all the analyzed data sets utilized in this study are deidentified and publicly available, ensuring the protection of patient privacy and confidentiality.

### Age-period-cohort analysis

To evaluate the independent effects of age, period, and birth cohort on NPC incidence, we used Age-Period-Cohort (APC) models. For this purpose, we grouped age (from 20–24 years to 85 + years) and period (from 1991–1995 to 2016–2020) into five-year intervals and defined 19 birth cohorts ranging from 1904–1908 to 1994–1998. The APC models estimated the net drift, which represents the annual percentage change in the expected age-adjusted rate over time, and the local drift, which refers to the annual percentage change over time for a particular age group. Incidence rate ratios (RRs) with 95% confidence intervals were used to present birth cohort and period effects. The central calendar period and birth cohort were set as the reference points for calculation, and the incidence rate ratio was 1.0 [15, 16]. We used the National Cancer Institute's Age-Period-Cohort web tool to fit the APC models [15]. The significance of variables was tested using Wald tests. Separate analyses were conducted for each sex, and all statistical tests were two-sided with a significance level of 0.05.

### Projection

The Bayesian APC framework is a statistical approach that allows for estimating uncertainty in projections by incorporating prior knowledge, such as historical trends and expert opinions, into the model [17]. This method can provide a more robust and reliable projection of future trends. The integrated nested Laplace approximation (INLA) is a computational tool that can be used to fit Bayesian models, which are complex statistical models that cannot be estimated using traditional methods. INLA is faster and more accurate than Markov Chain Monte Carlo methods, commonly used for Bayesian modelling, making it ideal for large-scale projects like population projections [17]. Our projection algorithm used the Bayesian APC framework with INLA to project the incidence and new cases of NPC in Hong Kong from 2021 to 2030. We used the BAPC package in R to fit our model and perform the projections. This package allows for the estimation of the posterior distributions of model parameters and provides reliable uncertainty estimates for the projections [17–19]. The population estimates used in our projections were based on Hong Kong population projections derived from the United Nations Population Division's World Population Prospects.

### Decomposition

To compare the impact of demographic and epidemiologic changes on NPC incidence, we decomposed the changes in new NPC cases in Hong Kong between 1991 and each subsequent year from 1992 to 2030 into population growth, population ageing, and age-specific incidence rates (i.e. epidemiologic changes). Epidemiologic changes refer to differences in new NPC cases that cannot be attributed to population growth or ageing and are the result of both risk factors and healthcare [20]. We performed the decomposition using a validated algorithm that considers the effects of individual factors and their interactions. It is insensitive to the decomposition order and the choice of reference year [12, 13]. Details of decomposition and projection are given in the appendix S1 and S2. The R programming language, version 3.6.3, was used for data processing and analysis.

## Results

### Trends in the incidence of NPC

**Fig. 1:**
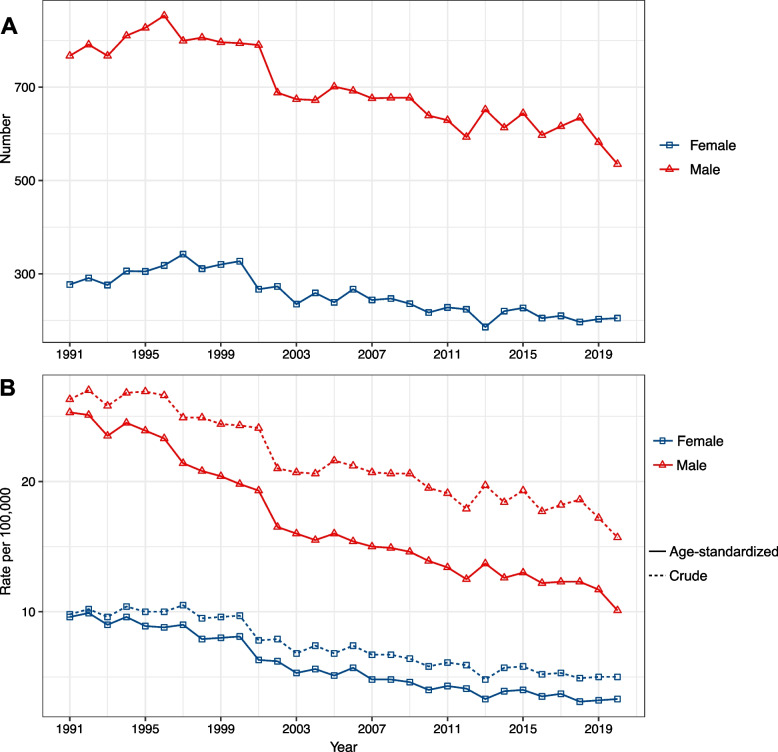
Changes in incidence rate and new cases of NPC in Hong Kong, 1991–2020. **A** New NPC cases. **B** Age-standardized and crude incidence rate

Over the 30-year period from 1991 to 2020, NPC incidence decreased for both sexes in Hong Kong. Among 28,653 diagnosed cases, 73.3% occurred in males and 26.7% in females. The number of new cases dropped from 767 and 277 in 1991 to 535 and 205 in 2020 for males and females, respectively (Fig. 1A). The crude and age-standardized NPC incidence rates declined for both sexes, with males experiencing a greater decline. The age-standardized incidence rate of NPC decreased from 25.3 and 9.6 per 100,000 populations in 1991 to 10.1 and 3.3 per 100,000 populations in 2020 for males and females, respectively (Fig. 1B).

### APC modelling

**Fig. 2:**
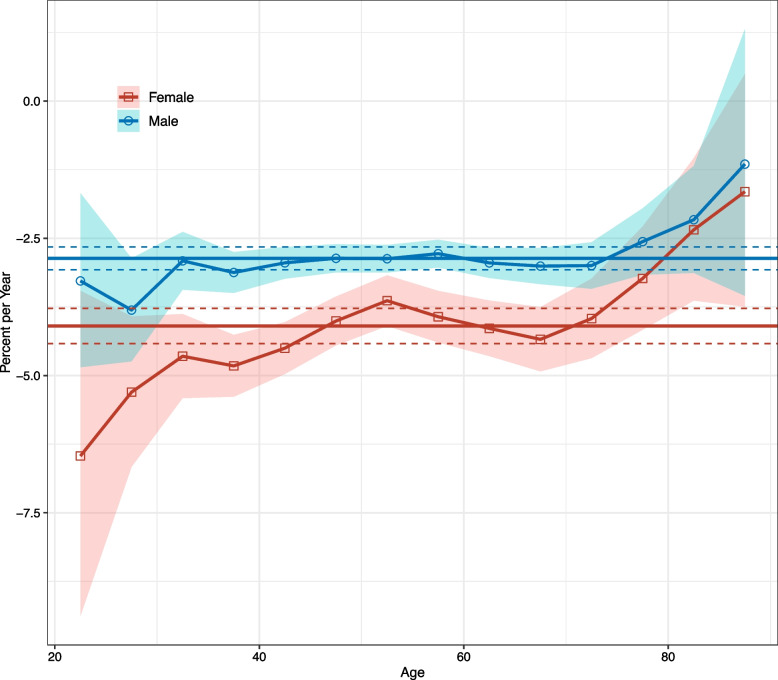
Local drifts with net drift values for NPC incidence in Hong Kong from 1991 to 2020. Shaded areas indicate 95% confidence interval

The net drifts showed statistically significant downward trends in NPC incidence, decreasing by -2.86% (95% confidence interval [CI]: -3.07% to -2.66%) per year for males and -4.10% (95% CI: -4.42% to -3.78%) per year for females over the entire period (Fig. 2). Local drift, which reflects changes in age-specific incidence trends, declined in both sexes at all ages, though 95% CIs were wide at both ends of the age group (Fig. 2).

**Fig. 3:**
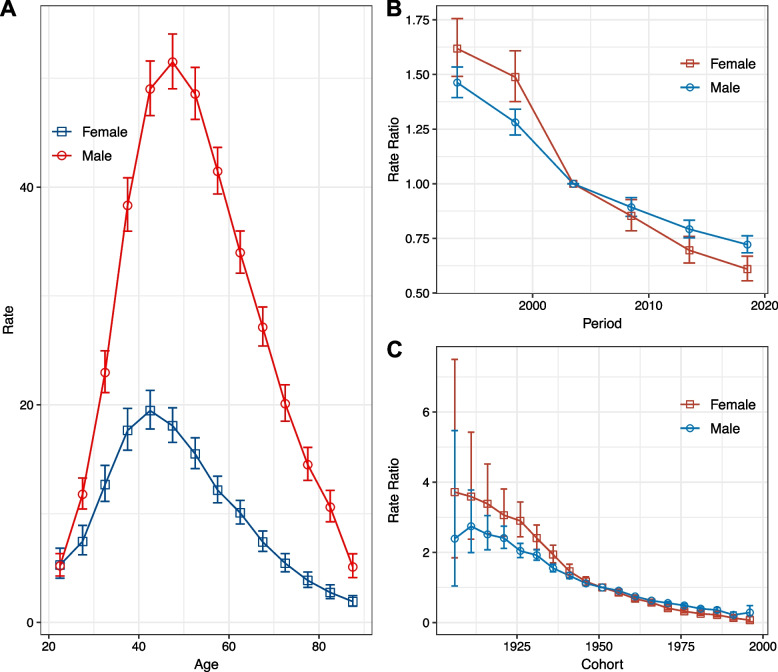
Age, calendar period, and birth cohort effects with the corresponding 95% confidence intervals on NPC incidence rates by sex, Hong Kong, 1991–2020. **A** Longitudinal curves of fitted age-specific rates in reference cohort adjusted for period effects; **B** Rate ratios in each period relative to the reference period, adjusted for age and non-linear cohort effects; **C** Rate ratios in each cohort relative to reference cohort, adjusted for age and non-linear period effects

After adjusting for period deviations, the incidence of NPC increased with age, peaked around 40–50, and then declined. Moreover, males had a significantly higher incidence rate than females, particularly in the high-incidence age groups (Fig. 3A).

Period rate ratios for both sexes showed patterns of monotonic decline, with females experiencing a more significant decline, indicating a more pronounced period effect (Fig. 3B). Cohort rate ratios also decreased monotonically for both sexes (Fig. 3C). The Wald test showed that the primary estimable functions were statistically significant for both sexes (Table S1). However, there was no statistical difference between local drift and net drift for males, indicating that the decline pattern per age group was not significantly different for males.

### Projection

**Fig. 4:**
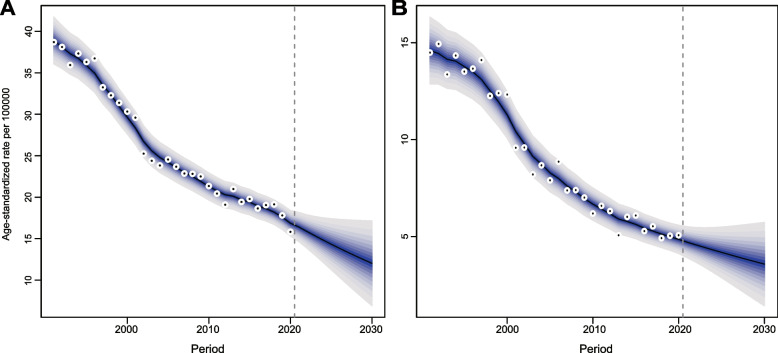
Trends and projected incidence rates for NPC in Hong Kong. **A** For Males; **B** For females. Dots represent fitted points. Data in the right of the dashed line were projected data. Each lighter shade of blue represents an additional 10% CI

New NPC cases are expected to fall from 535 to 436 for males and 205 to 179 for females between 2020 and 2030 (Tables S2, S3). Age-standardized incidence rates are also projected to decline for both sexes (Fig. 4).

### Decomposition

**Fig. 5:**
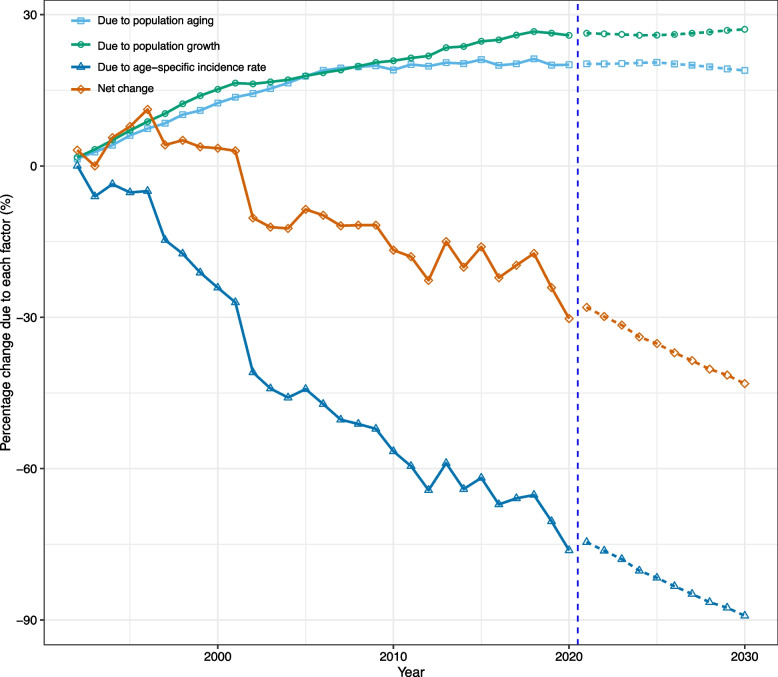
Contribution of changes in population aging, population growth, and age-specific incidence rate to changes in incident cases from 1992 to 2030 for Hong Kong males, using 1991 as the reference year. Data in the right of the blue dashed line were the decomposition based on the projected data

**Fig. 6:**
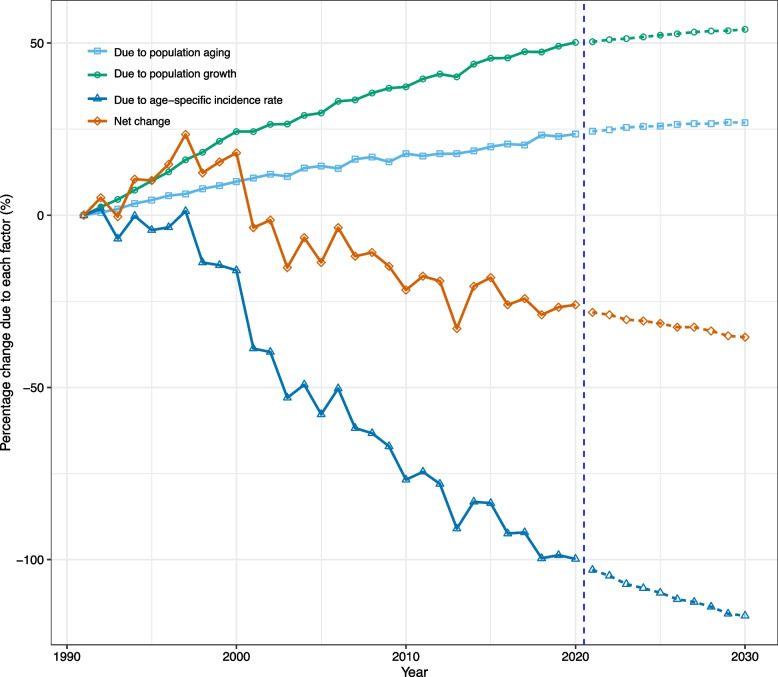
Contribution of changes in population aging, population growth, and age-specific incidence rate to changes in incident cases from 1992 to 2030 for Hong Kong females, using 1991 as the reference year. Data in the right of the blue dashed line were the decomposition based on the projected data

We found that, although population growth and ageing contributed to an increase in NPC cases, epidemiologic changes offset this increase, resulting in an encouraging downward trend in NPC incidence for both sexes. Specifically, between 1990 and 2020, the number of NPC cases in Hong Kong decreased by 30.2% (-232 cases) for males and 26.0% (-72 cases) for females. The increase in NPC cases for both sexes was primarily driven by demographic factors, with population ageing contributing to 20.1% and 23.6% increases for males and females, respectively, while population growth accounted for 25.9% and 50.2% increases for males and females, respectively. However, epidemiologic changes were responsible for a significant decrease in NPC cases, accounting for -76.2% and -99.8% of the difference for males and females, respectively (Figs. 5, 6; Tables S4, S5).

Our projections and decomposition analysis demonstrated that epidemiologic factors would remain the key driving force behind the decreasing trend of new NPC cases in Hong Kong. By 2030, we estimate that the number of NPC cases among males and females will be reduced by 43.2% (331 cases) and 35.4% (98 cases), respectively, compared to the figures from 1990 (Figs. 5, 6; Tables S4, S5).

## Discussion

This study represents the first investigation of the long-term incidence trends of NPC in Hong Kong over the past thirty years, as well as the underlying factors driving these trends. Our findings indicate a significant decline in the incidence of NPC, which can be attributed primarily to a reduction in both period and cohort risk of developing the disease. Despite an increase in NPC incidence due to population ageing and growth, the progress made in NPC prevention in Hong Kong has offset this effect. The study underscores the importance of ongoing epidemiological surveillance, clinical research, and implementing preventive measures to manage NPC.

The high incidence of NPC in Hong Kong may be attributed to various factors, including genetic predisposition, dietary habits and lifestyle, and their complex interactions [1, 21]. The observed decline in period and cohort effects in our study reflects changes in demographic and environmental factors. Hong Kong's economic development and adoption of a Western lifestyle have resulted in changes in dietary habits, with a decrease in the consumption of Cantonese-style salted fish, which is strongly associated with an elevated risk of NPC [21, 22]. Regularly consuming fresh fruits and vegetables, particularly during childhood, has been associated with a reduced risk of NPC [21–26]. On the other hand, cigarette smoking and occupational exposure to fumes, smoke, dust or chemicals are associated with an increased risk of NPC [1, 9]. However, Hong Kong's economic transition and effective smoking cessation programs have reduced exposure to these risk factors in recent birth cohorts. Notably, the smoking prevalence rate in Hong Kong has steadily declined from 23.3% in the early 1980s to 9.5% in 2021 [27]. These factors likely contribute to the downward trend in cohort risk of developing NPC and contribute to the observed period effect.

In the past 30 years, Hong Kong's population has increased by 1.5 million, with many people migrating from mainland China, where NPC is less common [21]. Consequently, the genetic makeup of the Hong Kong population has gradually evolved, which may partially account for the observed period effect. However, disentangling the period and cohort effects in real-world scenarios can be challenging, as different cohorts are born at different periods. Period effects tend to impact specific age groups differently when simultaneously affecting all age groups [15, 28]. Unfortunately, our study could not evaluate the role of risk factors in the changes in NPC incidence, as such data was not included in our data source.

Despite a pronounced downward trend in the age-standardized incidence rate of NPC in Hong Kong, the reduction in new cases is less pronounced due to the impact of demographic changes. The ageing of the population has led to an increase in the median age of the Hong Kong population from 29.9 years in 1988 to 45.3 years in 2018, resulting in a greater number of people aged 40–50 years with a high incidence of NPC [29]. Although epidemiologic changes have significantly decreased the number of new NPC cases in Hong Kong, the impact of population ageing and growth has slowed the decline. As fertility rates decline and life expectancy increases, the population ageing in Hong Kong will become more pronounced, potentially becoming a major contributor of the NPC cases.

Our findings align with the general downward trend in NPC incidence reported in previous epidemiologic studies [1, 23]. However, our study stands out by uncovering the underlying reasons for this trend, particularly the decline in cohort and period effects of NPC incidence. Notably, we quantitatively analyzed the contribution of demographic and epidemiological factors to changes in the number of NPC cases. These findings emphasize the consistent decrease in NPC incidence and underscore the significance of comprehending the interplay between demographic and epidemiological factors in shaping disease trends. Policymakers and public health professionals can leverage this information to design effective interventions for NPC prevention and control.

Several limitations are evident in our study. First, the lack of information on NPC subtypes in the HKCaR dataset limits our ability to examine the incidence trends of different pathological subtypes. Second, we lack information on patient risk factor exposure, such as dietary habits and information on smoking and alcohol consumption. Knowing this information would allow us to better assess the reasons for the decline in incidence and assist us in modifying our analysis. Third, the population structure and size of Hong Kong were derived from UN World Population Prospects, which may have introduced biases and underestimated the extent of ageing, potentially leading to variations in the projections. Finally, the use of different modelling approaches can introduce bias into our estimates.

## Conclusions

In conclusion, this study provides evidence that the decline in NPC incidence in Hong Kong was mainly due to epidemiologic changes that affected both the period and cohort risks of developing NPC. However, demographic changes such as population ageing and growth have lessened the effect of epidemiologic changes. Further investigation and epidemiologic analysis are needed to understand better the disease trends and potential risk factors contributing to NPC incidence in Hong Kong.

### Supplementary Information

**Additional file 1: Table S1. **Wald Chi-square tests for estimable parameters in the APC model. **Table S2.  **Estimated age-specific NPC cases for Hong Kong males from 1991 to 2030. **Table S3. **Estimated age-specific NPC cases for Hong Kong females from 1991 to 2030. **Table S4. **Contribution of changes in population aging, population growth, and age-specific incidence rate to the net change of NPC cases for Hong Kong males from 1992 to 2030. **Table S5. **Contribution of changes in population aging, population growth, and age-specific incidence rate to the net change of NPC cases for Hong Kong females from 1992 to 2030. 

## Data Availability

The datasets generated and analysed during the current study are available in the Hong Kong Cancer Registry: http://www3.ha.org.hk/cancereg/allages.asp.
