# Supplementary material for: Changing patterns of nasopharyngeal carcinoma incidence in Hong Kong: a 30-year analysis and future projections
Source: BMC Cancer. 2023 Aug 16;23:761. doi: 10.1186/s12885-023-11296-1 (PMC10429092; doi:10.1186/s12885-023-11296-1)
Supplement: Supplementary file 1 — Additional file 1: Table S1. Wald Chi-square tests for estimable parameters in the APC model. Table S2. Estimated age-specific NPC cases for Hong Kong males from 1991 to 2030. Table S3. Estimated age-specific NPC cases for Hong Kong females from 1991 to 2030. Table S4. Contribution of changes in population aging, population growth, and age-specific incidence rate to the net change of NPC cases for Hong Kong males from 1992 to 2030. Table S5. Contribution of changes in population aging, population growth, and age-specific incidence rate to the net change of NPC cases for Hong Kong females from 1992 to 2030. [file 12885_2023_11296_MOESM1_ESM.docx]

**SUPPLEMENTARY MATERIAL**

**Table of content**

**The decomposition method**

**Projection of future incidence**

**Table S1. Wald Chi-square tests for estimable parameters in the APC model**

**Table S2. Estimated age-specific NPC cases for Hong Kong males from 1991 to 2030.**

**Table S3. Estimated age-specific NPC cases for Hong Kong females from 1991 to 2030.**

**Table S4. Contribution of changes in population aging, population growth, and age-specific incidence rate to the net change of NPC cases for Hong Kong males from 1992 to 2030.**

**Table S5. Contribution of changes in population aging, population growth, and age-specific incidence rate to the net change of NPC cases for Hong Kong females from 1992 to 2030.**

**The decomposition method**

The population decomposition algorithm has been described in detail in the papers by Cheng et al. [1,2].

Briefly, take the difference in newly diagnosed NPC cases between 1991 and 2020 in Hong Kong. We can decompose the net change of incident cases into the contribution of three factors: population growth, population aging, and age-specific incidence rate.

The age groups were divided using 5-year increments from 30-34 years to 85 plus (we included older people aged ≥85 years as the 85-89 years age group, which was recorded as only one group in the database). Let *d_ij_*, *n_ij_*, *m_ij_* and *s_ij_* denote the incident cases, population size, age-specific rate of incidence, and population proportion in the *i*^th^ age group of the year *j*, respectively, (*i* = 1, 2, …,12; *j* = 1, 2). Let *D*_1_ and *D*_2_, *N*_1_ and *N*_2_, *P*_1_ and *P*_2_ represent the total incident cases, population size, and crude rate of incidence in 1990 and 2020.

Using *M_p_*, *M_a,_* and *M_m_* to represent the main effects of the changes in population size, age structure, and incidence rate, and *I_pa_*, *I_pm_*, *I_am,_* and *I_pam_* to represent their two-way and three-way interactions, respectively. In the case of 1991 as the reference year, these terms are calculated as follows:

$M_{p}=\sum_{i=1}^{12} {{\left( N_{2}-N_{1} \right)s}_{i1}m}_{i1}$

$M_{a}=\sum_{i=1}^{12} N_{1}\left( s_{i2}-s_{i1} \right)m_{i1}$

$M_{m}=\sum_{i=1}^{12} {N_{1}s}_{i1}\left( m_{i2}-m_{i1} \right)$

$I_{pa}=\sum_{i=1}^{12} \left( N_{2}-N_{1} \right)\left( s_{i2}-s_{i1} \right)m_{i1}$

$I_{pm}=\sum_{i=1}^{12} \left( N_{2}-N_{1} \right)s_{i1}\left( m_{i2}-m_{i1} \right)$

$I_{am}=\sum_{i=1}^{12} N_{1}\left( s_{i2}-s_{i1} \right)\left( m_{i2}-m_{i1} \right)$

$I_{pam}=\sum_{i=1}^{12} \left( N_{2}-N_{1} \right)\left( s_{i2}-s_{i1} \right)\left( m_{i2}-m_{i1} \right)$

Here, a simplification needs to be made, assuming that the interactions are equally distributed, then the contribution of the three factors can be calculated as follows:

$A{=M}_{a}+½I_{am}+½I_{pa}+⅓I_{pam}$

$P{=M}_{p}+½I_{pm}+½I_{pa}+⅓I_{pam}$

$M{=M}_{m}+½I_{pm}+½I_{am}+⅓I_{pam}$

Here, *A* represents the contribution of population aging, *P* represents the contribution of population growth, *M* represents the contribution of the age-specific death rate, and net change represents total change. The contribution of each factor divided by *D_1_* and multiplied by 100 is the percentage of the respective contribution.

**References**

1. Cheng X, Yang Y, Schwebel DC, Liu Z, Li L, Cheng P et al. Population ageing and mortality during 1990-2017: A global decomposition analysis. PLoS Med 2020;17:e1003138.

2. Cheng X, Tan L, Gao Y, Yang Y, Schwebel DC , Hu G. A new method to attribute differences in total deaths between groups to population size, age structure and age-specific mortality rate. PLoS One 2019;14:e0216613.

**Projection of future incidence**

The future incident cases of NPC in Hong Kong were projected using the Bayesian age-period-cohort analysis with integrated nested Laplace approximations (INLA). The Bayesian approach attributes separate effects to age, period and cohort, and extrapolates these effects to make projections. It does not depend on strong parametric assumptions like the classical approach. It is the only current method to achieve nonarbitrary and sensible projections.

Based on the expectation that adjacent effects in time might be similar, the Bayesian inference in the age-period-cohort model applies the second-order random walk for smoothing priors of age, period, and cohort effects and project posterior mortality rates. According to this model, each point of effect is predicted by linear extrapolation from its two immediate predecessors, plus a random variance from a normal distribution with a mean zero. The INLA is used with this Bayesian age-period-cohort model to approximate the marginal posterior distributions avoiding any mixing and convergence issues introduced by Markov chain Monte Carlo sampling techniques traditionally used in the Bayesian approach. The Bayesian age-period-cohort analysis was conducted by R-package BAPC (version 0.0.34).

We prepared age-specific incident cases of NPC (from 1991 to 2020) and Hong Kong population data (from 1991 to 2030), followed by a 10-year (from 2021 to 2030) retrospective projection using the BAPC function in the R package BAPC.

**References**

1. Riebler, A. and L. Held, Projecting the future burden of cancer: Bayesian age-period-cohort analysis with integrated nested Laplace approximations. Biometrical Journal, 2017. 59(3): p. 531-549.

2. Jacobs, D., et al., Assessment of Age, Period, and Birth Cohort Effects and Trends in Merkel Cell Carcinoma Incidence in the United States. Jama Dermatology, 2021. 157(1): p. 59-65.

**Table S1. Wald Chi-square tests for estimable parameters in the APC model**

| Null Hypothesis | Males | | Females | |
| --- | --- | --- | --- | --- |
|  | Chi-squre | P-value | Chi-squre | P-value |
| NetDrift = 0 | 706.3 | <0.001 | 602.3 | <0.001 |
| All Age Deviations = 0 | 2223.0 | <0.001 | 584.3 | <0.001 |
| All Period RR = 1 | 756.8 | <0.001 | 651.1 | <0.001 |
| All Cohort RR = 1 | 1297.9 | <0.001 | 870.2 | <0.001 |
| All Local Drifts = Net Drift | 20.7 | 0.11 | 28.3 | 0.01 |

**Table S2. Estimated age-specific NPC cases for Hong Kong males from 1991 to 2030.**

| Year |  | Number of age-specific NPC cases | | | | | | | | | | | | | |
| --- | --- | --- | --- | --- | --- | --- | --- | --- | --- | --- | --- | --- | --- | --- | --- |
|  | 20-24 | 25-29 | 30-34 | 35-39 | 40-44 | 45-49 | 50-54 | 55-59 | 60-64 | 65-69 | 70-74 | 75-79 | 80-84 | 85+ | Total |
| 1991 | 6 | 26 | 60 | 90 | 117 | 86 | 107 | 98 | 75 | 47 | 29 | 16 | 8 | 2 | 767 |
| 1992 | 4 | 32 | 49 | 104 | 110 | 102 | 70 | 88 | 98 | 74 | 33 | 25 | 1 | 1 | 791 |
| 1993 | 4 | 17 | 59 | 105 | 116 | 109 | 80 | 82 | 91 | 58 | 30 | 8 | 7 | 1 | 767 |
| 1994 | 5 | 17 | 48 | 97 | 120 | 117 | 94 | 95 | 97 | 63 | 35 | 15 | 5 | 2 | 810 |
| 1995 | 9 | 8 | 49 | 115 | 134 | 116 | 81 | 98 | 87 | 65 | 39 | 16 | 10 | 0 | 827 |
| 1996 | 10 | 18 | 40 | 114 | 141 | 124 | 88 | 98 | 78 | 66 | 39 | 24 | 10 | 3 | 853 |
| 1997 | 8 | 22 | 43 | 108 | 134 | 126 | 86 | 77 | 81 | 49 | 30 | 27 | 4 | 4 | 799 |
| 1998 | 5 | 19 | 44 | 100 | 147 | 130 | 103 | 68 | 61 | 65 | 42 | 16 | 5 | 1 | 806 |
| 1999 | 7 | 20 | 35 | 91 | 138 | 116 | 109 | 71 | 77 | 66 | 32 | 23 | 9 | 2 | 796 |
| 2000 | 6 | 15 | 41 | 97 | 149 | 130 | 96 | 66 | 67 | 53 | 41 | 16 | 13 | 4 | 794 |
| 2001 | 5 | 19 | 39 | 69 | 150 | 115 | 128 | 69 | 63 | 60 | 41 | 17 | 13 | 2 | 790 |
| 2002 | 5 | 8 | 27 | 69 | 116 | 111 | 119 | 65 | 58 | 47 | 35 | 16 | 8 | 4 | 688 |
| 2003 | 3 | 19 | 27 | 58 | 100 | 136 | 98 | 76 | 43 | 44 | 39 | 18 | 7 | 6 | 674 |
| 2004 | 2 | 7 | 21 | 64 | 109 | 131 | 99 | 67 | 53 | 47 | 43 | 17 | 9 | 3 | 672 |
| 2005 | 7 | 13 | 28 | 57 | 98 | 145 | 117 | 73 | 38 | 55 | 33 | 25 | 11 | 1 | 701 |
| 2006 | 5 | 5 | 31 | 51 | 93 | 129 | 130 | 90 | 35 | 45 | 34 | 28 | 10 | 6 | 692 |
| 2007 | 3 | 8 | 20 | 58 | 78 | 121 | 137 | 97 | 43 | 39 | 37 | 21 | 10 | 4 | 676 |
| 2008 | 5 | 8 | 25 | 51 | 77 | 133 | 130 | 85 | 67 | 36 | 25 | 17 | 15 | 3 | 677 |
| 2009 | 5 | 9 | 26 | 38 | 90 | 98 | 128 | 100 | 64 | 34 | 42 | 33 | 8 | 2 | 677 |
| 2010 | 1 | 8 | 27 | 51 | 75 | 100 | 113 | 87 | 72 | 39 | 40 | 14 | 8 | 4 | 639 |
| 2011 | 2 | 8 | 28 | 47 | 56 | 92 | 133 | 75 | 75 | 35 | 29 | 27 | 20 | 2 | 629 |
| 2012 | 2 | 8 | 25 | 35 | 60 | 88 | 109 | 100 | 73 | 37 | 23 | 19 | 11 | 3 | 593 |
| 2013 | 2 | 13 | 21 | 45 | 71 | 103 | 105 | 101 | 72 | 39 | 24 | 31 | 18 | 7 | 652 |
| 2014 | 3 | 5 | 18 | 43 | 62 | 90 | 93 | 104 | 74 | 54 | 27 | 24 | 11 | 5 | 613 |
| 2015 | 2 | 8 | 26 | 35 | 53 | 72 | 94 | 132 | 74 | 77 | 31 | 21 | 14 | 5 | 644 |
| 2016 | 3 | 7 | 29 | 33 | 54 | 80 | 92 | 103 | 82 | 49 | 27 | 19 | 10 | 9 | 597 |
| 2017 | 5 | 1 | 21 | 40 | 68 | 74 | 89 | 107 | 88 | 50 | 37 | 15 | 15 | 6 | 616 |
| 2018 | 4 | 11 | 16 | 40 | 44 | 80 | 93 | 97 | 103 | 69 | 41 | 18 | 12 | 6 | 634 |
| 2019 | 3 | 5 | 18 | 34 | 63 | 71 | 79 | 110 | 74 | 51 | 41 | 13 | 15 | 5 | 582 |
| 2020 | 1 | 8 | 20 | 27 | 46 | 63 | 58 | 78 | 90 | 70 | 36 | 21 | 10 | 7 | 535 |
| 2021 | 2 | 7 | 16 | 33 | 50 | 63 | 71 | 84 | 86 | 62 | 40 | 18 | 12 | 8 | 552 |
| 2022 | 2 | 6 | 16 | 32 | 49 | 61 | 68 | 78 | 83 | 64 | 40 | 20 | 11 | 8 | 538 |
| 2023 | 2 | 6 | 15 | 31 | 48 | 58 | 66 | 72 | 80 | 66 | 41 | 22 | 10 | 8 | 525 |
| 2024 | 1 | 5 | 15 | 29 | 46 | 56 | 64 | 67 | 76 | 67 | 41 | 23 | 9 | 8 | 507 |
| 2025 | 1 | 5 | 14 | 28 | 45 | 55 | 62 | 63 | 72 | 67 | 43 | 25 | 9 | 8 | 497 |
| 2026 | 1 | 5 | 13 | 28 | 43 | 53 | 60 | 60 | 68 | 66 | 44 | 25 | 10 | 7 | 483 |
| 2027 | 1 | 4 | 13 | 27 | 42 | 52 | 58 | 58 | 63 | 64 | 45 | 26 | 11 | 7 | 471 |
| 2028 | 1 | 4 | 12 | 26 | 40 | 51 | 56 | 56 | 59 | 61 | 47 | 26 | 12 | 7 | 458 |
| 2029 | 2 | 4 | 11 | 26 | 39 | 50 | 54 | 55 | 55 | 59 | 47 | 27 | 13 | 7 | 449 |
| 2030 | 2 | 3 | 11 | 25 | 38 | 48 | 52 | 53 | 52 | 56 | 47 | 28 | 14 | 7 | 436 |

**Table S3. Estimated age-specific NPC incident cases for Hong Kong females from 1991 to 2030.**

| Year |  | Number of age-specific NPC cases | | | | | | | | | | | | | |
| --- | --- | --- | --- | --- | --- | --- | --- | --- | --- | --- | --- | --- | --- | --- | --- |
|  | 20-24 | 25-29 | 30-34 | 35-39 | 40-44 | 45-49 | 50-54 | 55-59 | 60-64 | 65-69 | 70-74 | 75-79 | 80-84 | 85+ | Total |
| 1991 | 5 | 8 | 20 | 38 | 41 | 24 | 28 | 35 | 34 | 23 | 12 | 3 | 5 | 1 | 277 |
| 1992 | 2 | 11 | 25 | 41 | 32 | 32 | 33 | 41 | 30 | 14 | 12 | 11 | 4 | 3 | 291 |
| 1993 | 4 | 13 | 29 | 32 | 40 | 31 | 23 | 28 | 26 | 19 | 17 | 9 | 2 | 3 | 276 |
| 1994 | 6 | 17 | 28 | 41 | 42 | 45 | 21 | 31 | 27 | 24 | 12 | 6 | 6 | 0 | 306 |
| 1995 | 6 | 12 | 34 | 47 | 52 | 30 | 28 | 14 | 26 | 32 | 13 | 4 | 4 | 3 | 305 |
| 1996 | 7 | 9 | 28 | 47 | 59 | 36 | 27 | 20 | 26 | 21 | 18 | 10 | 8 | 2 | 318 |
| 1997 | 7 | 18 | 43 | 38 | 50 | 49 | 23 | 21 | 25 | 32 | 15 | 10 | 8 | 3 | 342 |
| 1998 | 6 | 11 | 22 | 51 | 62 | 38 | 24 | 16 | 25 | 24 | 18 | 8 | 3 | 3 | 311 |
| 1999 | 4 | 10 | 29 | 47 | 55 | 49 | 33 | 17 | 27 | 22 | 14 | 7 | 5 | 1 | 320 |
| 2000 | 5 | 7 | 21 | 56 | 50 | 53 | 35 | 22 | 22 | 24 | 15 | 11 | 3 | 3 | 327 |
| 2001 | 1 | 6 | 11 | 45 | 48 | 39 | 35 | 18 | 13 | 24 | 9 | 10 | 4 | 4 | 267 |
| 2002 | 3 | 6 | 10 | 37 | 50 | 39 | 42 | 17 | 19 | 18 | 12 | 8 | 7 | 5 | 273 |
| 2003 | 3 | 7 | 17 | 35 | 31 | 41 | 34 | 22 | 11 | 14 | 7 | 8 | 4 | 1 | 235 |
| 2004 | 1 | 5 | 14 | 34 | 42 | 44 | 41 | 22 | 16 | 9 | 13 | 10 | 4 | 4 | 259 |
| 2005 | 6 | 4 | 10 | 18 | 43 | 48 | 31 | 25 | 15 | 14 | 9 | 10 | 3 | 3 | 239 |
| 2006 | 2 | 6 | 17 | 23 | 38 | 48 | 37 | 26 | 29 | 14 | 15 | 6 | 4 | 2 | 267 |
| 2007 | 0 | 4 | 13 | 19 | 40 | 44 | 38 | 23 | 10 | 12 | 14 | 11 | 10 | 6 | 244 |
| 2008 | 2 | 2 | 11 | 19 | 42 | 48 | 35 | 24 | 17 | 9 | 15 | 12 | 6 | 5 | 247 |
| 2009 | 2 | 4 | 12 | 27 | 33 | 32 | 34 | 18 | 20 | 7 | 19 | 20 | 4 | 4 | 236 |
| 2010 | 2 | 0 | 8 | 23 | 30 | 45 | 31 | 27 | 15 | 5 | 10 | 8 | 7 | 6 | 217 |
| 2011 | 3 | 6 | 11 | 18 | 29 | 44 | 30 | 28 | 14 | 14 | 9 | 7 | 8 | 7 | 228 |
| 2012 | 1 | 6 | 9 | 18 | 29 | 34 | 34 | 27 | 25 | 10 | 10 | 13 | 6 | 2 | 224 |
| 2013 | 0 | 3 | 11 | 8 | 26 | 23 | 30 | 30 | 18 | 9 | 12 | 4 | 5 | 7 | 186 |
| 2014 | 1 | 9 | 13 | 17 | 27 | 27 | 36 | 31 | 23 | 13 | 6 | 5 | 5 | 7 | 220 |
| 2015 | 1 | 1 | 12 | 25 | 23 | 30 | 29 | 34 | 29 | 19 | 10 | 7 | 3 | 4 | 227 |
| 2016 | 0 | 4 | 7 | 17 | 22 | 20 | 32 | 29 | 33 | 21 | 6 | 8 | 4 | 2 | 205 |
| 2017 | 2 | 6 | 12 | 20 | 27 | 21 | 34 | 33 | 14 | 22 | 8 | 2 | 3 | 6 | 210 |
| 2018 | 0 | 1 | 9 | 17 | 15 | 37 | 33 | 29 | 21 | 12 | 10 | 4 | 1 | 8 | 197 |
| 2019 | 0 | 3 | 11 | 13 | 27 | 23 | 29 | 32 | 31 | 12 | 10 | 4 | 4 | 4 | 203 |
| 2020 | 2 | 1 | 10 | 19 | 18 | 27 | 31 | 27 | 28 | 16 | 14 | 4 | 4 | 4 | 205 |
| 2021 | 1 | 3 | 7 | 15 | 21 | 24 | 26 | 29 | 26 | 19 | 12 | 6 | 4 | 6 | 199 |
| 2022 | 1 | 3 | 7 | 14 | 21 | 24 | 25 | 28 | 26 | 19 | 13 | 6 | 4 | 6 | 197 |
| 2023 | 1 | 2 | 6 | 13 | 20 | 23 | 25 | 26 | 27 | 20 | 13 | 7 | 4 | 6 | 193 |
| 2024 | 1 | 2 | 6 | 12 | 20 | 23 | 24 | 25 | 27 | 20 | 14 | 8 | 4 | 6 | 192 |
| 2025 | 1 | 2 | 6 | 12 | 19 | 22 | 24 | 24 | 26 | 21 | 14 | 9 | 4 | 6 | 190 |
| 2026 | 1 | 2 | 5 | 11 | 19 | 22 | 23 | 23 | 26 | 21 | 15 | 9 | 4 | 6 | 187 |
| 2027 | 1 | 2 | 5 | 11 | 18 | 22 | 23 | 23 | 25 | 22 | 15 | 10 | 4 | 6 | 187 |
| 2028 | 1 | 2 | 5 | 10 | 17 | 22 | 23 | 22 | 24 | 22 | 16 | 10 | 5 | 5 | 184 |
| 2029 | 1 | 1 | 4 | 10 | 16 | 22 | 22 | 22 | 23 | 22 | 16 | 10 | 6 | 5 | 180 |
| 2030 | 1 | 1 | 4 | 9 | 16 | 21 | 22 | 22 | 22 | 22 | 17 | 11 | 6 | 5 | 179 |

**Table S4. Contribution of changes in population aging, population growth, and age-specific incidence rate to the net change of NPC cases for Hong Kong males from 1992 to 2030.** 1991 was used as the reference year.

| year | Population aging(%) | Population growth(%) | Epidemiological change(%) | Net change(%) |
| --- | --- | --- | --- | --- |
| 1992 | 11 (1.4) | 13 (1.7) | 0 (0.0) | 24 (3.1) |
| 1993 | 21 (2.8) | 25 (3.3) | -46 (-6.1) | 0 (0.0) |
| 1994 | 32 (4.1) | 39 (5.1) | -28 (-3.6) | 43 (5.6) |
| 1995 | 46 (6.1) | 54 (7.0) | -40 (-5.3) | 60 (7.8) |
| 1996 | 57 (7.4) | 67 (8.8) | -38 (-5.0) | 86 (11.2) |
| 1997 | 65 (8.5) | 80 (10.4) | -113 (-14.7) | 32 (4.2) |
| 1998 | 78 (10.2) | 95 (12.3) | -133 (-17.4) | 39 (5.1) |
| 1999 | 84 (11.0) | 107 (13.9) | -162 (-21.1) | 29 (3.8) |
| 2000 | 96 (12.5) | 116 (15.2) | -185 (-24.2) | 27 (3.5) |
| 2001 | 104 (13.6) | 126 (16.4) | -207 (-27.0) | 23 (3.0) |
| 2002 | 110 (14.4) | 125 (16.3) | -314 (-40.9) | -79 (-10.3) |
| 2003 | 118 (15.3) | 128 (16.7) | -338 (-44.1) | -93 (-12.1) |
| 2004 | 126 (16.5) | 131 (17.1) | -352 (-45.9) | -95 (-12.4) |
| 2005 | 136 (17.8) | 137 (17.8) | -339 (-44.2) | -66 (-8.6) |
| 2006 | 145 (18.9) | 142 (18.5) | -362 (-47.2) | -75 (-9.8) |
| 2007 | 149 (19.4) | 146 (19.0) | -386 (-50.3) | -91 (-11.9) |
| 2008 | 151 (19.6) | 152 (19.8) | -392 (-51.2) | -90 (-11.7) |
| 2009 | 152 (19.9) | 157 (20.5) | -400 (-52.1) | -90 (-11.7) |
| 2010 | 146 (19) | 160 (20.9) | -434 (-56.6) | -128 (-16.7) |
| 2011 | 154 (20.1) | 164 (21.4) | -456 (-59.5) | -138 (-18.0) |
| 2012 | 152 (19.8) | 167 (21.8) | -493 (-64.3) | -174 (-22.7) |
| 2013 | 157 (20.5) | 180 (23.5) | -452 (-58.9) | -115 (-15.0) |
| 2014 | 156 (20.3) | 182 (23.7) | -491 (-64.1) | -154 (-20.1) |
| 2015 | 162 (21.1) | 190 (24.7) | -474 (-61.8) | -123 (-16.0) |
| 2016 | 153 (19.9) | 192 (25.0) | -515 (-67.1) | -170 (-22.2) |
| 2017 | 155 (20.3) | 199 (25.9) | -505 (-65.9) | -151 (-19.7) |
| 2018 | 163 (21.3) | 204 (26.6) | -500 (-65.2) | -133 (-17.3) |
| 2019 | 153 (20.0) | 202 (26.3) | -540 (-70.4) | -185 (-24.1) |
| 2020 | 154 (20.1) | 199 (25.9) | -584 (-76.2) | -232 (-30.2) |
| 2021 | 155 (20.2) | 202 (26.3) | -572 (-74.6) | -215 (-28) |
| 2022 | 155 (20.2) | 201 (26.2) | -585 (-76.3) | -229 (-29.9) |
| 2023 | 156 (20.3) | 200 (26.1) | -598 (-77.9) | -242 (-31.6) |
| 2024 | 157 (20.4) | 199 (25.9) | -615 (-80.2) | -260 (-33.9) |
| 2025 | 157 (20.5) | 199 (25.9) | -626 (-81.7) | -270 (-35.2) |
| 2026 | 155 (20.2) | 200 (26.1) | -639 (-83.3) | -284 (-37.0) |
| 2027 | 153 (20.0) | 202 (26.3) | -651 (-84.9) | -296 (-38.6) |
| 2028 | 151 (19.7) | 204 (26.6) | -663 (-86.5) | -309 (-40.3) |
| 2029 | 148 (19.2) | 206 (26.9) | -672 (-87.6) | -318 (-41.5) |
| 2030 | 145 (18.9) | 208 (27.1) | -684 (-89.2) | -331 (-43.2) |

**Table S5. Contribution of changes in population aging, population growth, and age-specific incidence rate to the net change of NPC cases for Hong Kong females from 1991 to 2030.** 1991 was used as the reference year.

| year | Population aging(%) | Population growth(%) | Epidemiological change(%) | Net change(%) |
| --- | --- | --- | --- | --- |
| 1992 | 2 (0.8) | 7 (2.4) | 5 (1.9) | 14 (5.1) |
| 1993 | 5 (1.8) | 13 (4.6) | -19 (-6.8) | -1 (-0.4) |
| 1994 | 9 (3.4) | 20 (7.3) | -1 (-0.2) | 29 (10.5) |
| 1995 | 12 (4.4) | 28 (10.0) | -12 (-4.3) | 28 (10.1) |
| 1996 | 16 (5.7) | 35 (12.6) | -10 (-3.5) | 41 (14.8) |
| 1997 | 17 (6.2) | 45 (16.1) | 3 (1.2) | 65 (23.5) |
| 1998 | 21 (7.7) | 51 (18.3) | -38 (-13.7) | 34 (12.3) |
| 1999 | 24 (8.6) | 59 (21.5) | -40 (-14.5) | 43 (15.5) |
| 2000 | 27 (9.8) | 67 (24.3) | -44 (-16) | 50 (18.1) |
| 2001 | 30 (10.8) | 67 (24.3) | -107 (-38.7) | -10 (-3.6) |
| 2002 | 33 (11.9) | 73 (26.4) | -110 (-39.7) | -4 (-1.4) |
| 2003 | 31 (11.3) | 73 (26.5) | -147 (-53) | -42 (-15.2) |
| 2004 | 38 (13.7) | 80 (29.0) | -136 (-49.2) | -18 (-6.5) |
| 2005 | 40 (14.3) | 82 (29.7) | -160 (-57.8) | -38 (-13.7) |
| 2006 | 38 (13.6) | 92 (33.1) | -139 (-50.3) | -10 (-3.6) |
| 2007 | 45 (16.3) | 93 (33.5) | -171 (-61.8) | -33 (-11.9) |
| 2008 | 47 (16.9) | 98 (35.5) | -175 (-63.3) | -30 (-10.8) |
| 2009 | 43 (15.5) | 102 (36.9) | -186 (-67.1) | -41 (-14.8) |
| 2010 | 49 (17.9) | 103 (37.3) | -213 (-76.8) | -60 (-21.7) |
| 2011 | 48 (17.2) | 110 (39.6) | -206 (-74.5) | -49 (-17.7) |
| 2012 | 50 (17.9) | 114 (41.0) | -216 (-78.0) | -53 (-19.1) |
| 2013 | 50 (17.9) | 111 (40.2) | -252 (-91.0) | -91 (-32.9) |
| 2014 | 52 (18.7) | 122 (43.9) | -230 (-83.2) | -57 (-20.6) |
| 2015 | 55 (19.9) | 126 (45.6) | -232 (-83.6) | -50 (-18.1) |
| 2016 | 57 (20.7) | 126 (45.7) | -256 (-92.4) | -72 (-26.0) |
| 2017 | 57 (20.4) | 132 (47.5) | -255 (-92.1) | -67 (-24.2) |
| 2018 | 65 (23.3) | 131 (47.4) | -276 (-99.6) | -80 (-28.9) |
| 2019 | 64 (22.9) | 136 (49.1) | -274 (-98.7) | -74 (-26.7) |
| 2020 | 65 (23.6) | 139 (50.2) | -276 (-99.8) | -72 (-26.0) |
| 2021 | 68 (24.4) | 140 (50.4) | -285 (-103.0) | -78 (-28.2) |
| 2022 | 69 (24.8) | 141 (51.0) | -290 (-104.7) | -80 (-28.9) |
| 2023 | 71 (25.5) | 142 (51.3) | -297 (-107.1) | -84 (-30.3) |
| 2024 | 71 (25.8) | 144 (51.8) | -300 (-108.3) | -85 (-30.7) |
| 2025 | 72 (25.9) | 145 (52.3) | -304 (-109.6) | -87 (-31.4) |
| 2026 | 73 (26.4) | 146 (52.7) | -309 (-111.5) | -90 (-32.5) |
| 2027 | 74 (26.6) | 147 (53.2) | -311 (-112.3) | -90 (-32.5) |
| 2028 | 74 (26.6) | 148 (53.5) | -315 (-113.7) | -93 (-33.6) |
| 2029 | 75 (27.0) | 149 (53.6) | -320 (-115.7) | -97 (-35.0) |
| 2030 | 75 (26.9) | 149 (54.0) | -322 (-116.3) | -98 (-35.4) |
